# A curious case of granulomatosis with polyangiitis: prostatic and skin involvement

**DOI:** 10.1093/rap/rkac033

**Published:** 2022-07-27

**Authors:** Laura Trives-Folguera, David Isenberg

**Affiliations:** Centre for Rheumatology, Hospital General Universitario Gregorio Marañón, Madrid, Spain; Centre for Rheumatology, University College Hospital London, London, UK

Key message

- Knowing the different forms of presentation of granulomatosis with polyangiitis is important to save the patient’s life.

Dear Editor, Granulomatosis with polyangiitis (GPA) is a systemic necrotizing vasculitis that affects small and medium-sized vessels and is often associated with cytoplasmic ANCA. It usually affects the upper and lower respiratory tract and the kidneys, which, if left untreated, can cause life-threatening organ damage [1]. Although they are the most commonly affected systems, GPA can also involve other organs, including the bladder or skin. Early diagnosis, based on clinical features, laboratory data with ANCA levels and subtype, and histopathology, is important for the timely initiation of immunosuppressive treatment [2]. We describe the rare case of a 66-year-old male diagnosed with GPA because of prostatitis, granulomatosis and cutaneous lesions.

A 66-year-old male was referred to the urology service in a community hospital in 2005 because he developed a bladder outflow obstruction and microscopic haematuria. Prostate biopsies revealed ‘inflammatory tissue’, hence he was prescribed CS treatment and was referred to an academic hospital for a second opinion. The biopsies were reviewed and showed ‘extensive ulcerative granulomatous tissue formation and acute and chronic inflammation; areas of micro-abscesses and foci of granulomatous inflammation with some appearing to be centred on vessel’. He developed an unusual cutaneous lesion on the proximal component of his fourth right finger, which was evaluated by dermatologist. In the letter of referral to our rheumatology department, we were told that the local pathologists had reviewed the biopsy and concluded that it showed evidence of vasculitis. A PR3 ANCA level of 29 UI/ml (normal: <2 UI/ml) was recorded. He was diagnosed with GPA and started immunosuppressive treatment with i.v. methylprednisolone and weekly MTX. He improved rapidly, going into remission from the skin involvement within 3 months. The cANCA became normal during the next 5 years. Given that he was in clinical and serological remission, MTX was stopped in 2012. A few months later, he developed urethral bleeding, and a new biopsy showed activity of the vasculitis that required immunosuppressive treatment with CSs and induction with i.v. CYC. MTX was subsequently reinitiated as maintenance treatment.

He remained well during the next 7 years and was able to reduce both MTX and prednisone doses gradually until their cessation in June 2019 and February 2020, respectively.

There was a delay in follow-up owing to the coronavirus disease 2019 pandemic, but he was seen again in our clinic in July 2021. Although feeling well in general, he had developed a lesion on his left temple since May 2021 (Fig. 1). An initial biopsy was reported to show non-specific inflammatory changes only, but a deeper one in September 2021 reported chronic inflammation and granulomatous inflammation. In addition, his cANCA increased from 0.6 to 3.1 UI/ml (normal now <2.9 UI/ml). Treatment with prednisone and AZA was prescribed, but he had to stop the latter because of intolerance, and he received two doses of rituximab in October 2021. When reviewed in the clinic in November 2021, he was feeling well and had partial resolution of the temple lesion, without any new symptoms. He has maintained his prednisolone but with a decreasing dose.

**Fig. 1:**
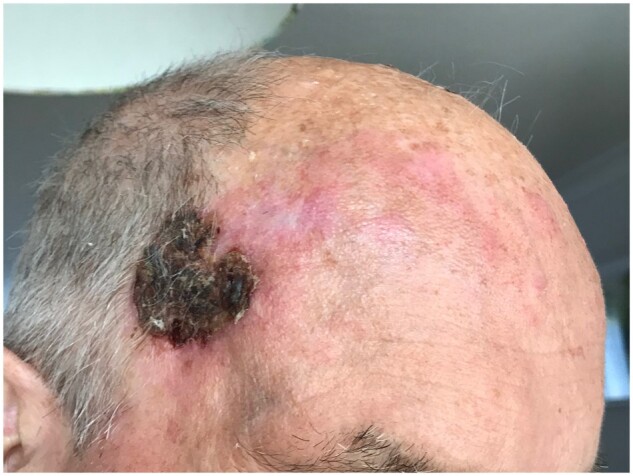
Ulcerative lesion in the left temple of the patient

This report describes a case of GPA presenting with prostatic involvement, which is unusual. The incidence of genitourinary symptoms in this disease is <1%, with few cases described in the literature [3, 4]. It is usually observed at disease onset and as part of generalized systemic disease, with isolated involvement of the urogenital tract being rare [5, 6]. Up to 18% of the cases described had isolated urogenital symptoms with a prior suspicion of malignancy, but later developed signs of generalized GPA. Prostatitis is the most common presentation (≤37% of cases), and the main symptoms are macroscopic haematuria, dysuria and obstructive symptoms [7]. The bladder is the second organ most affected (25% of cases), in which cytoscopy reveals a diffusely thickened bladder, with ulcerations and fibrosis [6, 7]. Furthermore, the skin involvement in our patient is not characteristic of GPA. As described by Micheletti *et al.* [8], the most frequent cutaneous manifestations in ANCA-associated vasculitis are petechiae/purpura, painful skin lesions and maculopapular rash. Skin involvement is correlated with systemic disease and cANCA positivity [8].

The combination of a single skin lesion and bladder involvement is most unusual in patients with GPA. Prostatic involvement is also rare, but in patients with unexplained prostatitis the possibility of this disease should be considered. Histopathological examination will often be the key to establish a diagnosis of GPA in patients with atypical organ involvement.

*Funding:* No specific funding was received from any bodies in the public, commercial or not-for-profit sectors to carry out the work described in this article.

*Disclosure statement:* All the authors declare no conflicts of interest.

*Consent:* Informed consent was provided for the publication of this paper.

## Data Availability

The authors declare that all relevant data supporting the findings of this case report are available within the article.
